# An Insight Into the Association of Sclerostin With Insulin Sensitivity and Glycemic Parameters in Male Indian Prediabetic and Diabetic Population

**DOI:** 10.7759/cureus.27123

**Published:** 2022-07-21

**Authors:** Praveen K Singh, Manisha Naithani, Monika Pathania, Anissa A Mirza, Sarama Saha

**Affiliations:** 1 Biochemistry, All India Institute of Medical Sciences, Rishikesh, Rishikesh, IND; 2 Internal Medicine, All India Institute of Medical Sciences, Rishikesh, Rishikesh, IND

**Keywords:** diabetes mellitus, glycemic parameters, spise index, type 2 diabetes, sclerostin

## Abstract

Background

Type 2 diabetes (T2D) is increasing day by day and creating a huge financial and social burden on the Indian population. Insulin resistance results in hyperglycemia, a condition that eventually causes prediabetes and Type 2 diabetes. The etiopathogenesis of T2D is still not clearly defined. Wnt signaling pathway is involved in pancreas development, islet function, insulin production, and secretion. Recent studies show that sclerostin, a Wnt signaling inhibitor, is associated with diabetes. The sclerostin level is altered as a function of race and ethnicity. However, no study has been conducted to observe the sclerostin level in prediabetic and diabetic individuals in the Indian population.

Objectives

The main objectives of the study are: to determine whether sclerostin is associated with glycemic parameters, serum insulin levels, insulin resistance/ sensitivity, beta-cell function, and adipose tissue insulin resistance (Adipo-IR).

Methods

This observational study was carried out at a tertiary care hospital, in Rishikesh, Uttarakhand, India. Individuals with T2D and prediabetes and healthy references were included in this study. Sclerostin and free fatty acids (FFA) were measured with the enzyme-linked immunosorbent assay (ELISA), and blood sugar, insulin, and glycated haemoglobin (HbA1c) were measured by the hexokinase, chemiluminescent, and chromatography methods, respectively. Messenger RNA (mRNA) was quantified by real-time polymerase chain reaction (PCR) using the SYBR Green protocol. Adipo-IR, homeostasis model assessment-estimated insulin resistance (HOMA-IR), homeostasis model assessment of β-cell function (HOMA-B), quantitative insulin sensitivity check index (QUICKI), and single point insulin sensitivity estimator (SPISE) indices were calculated.

Results

A total of 171 study participants were enrolled in type 2 diabetes, prediabetes, and controls groups, having 57 each in the group. There was a gradual increase in sclerostin levels from healthy [242.12(158.44)] to prediabetes [256.06(299.65)] and diabetes [465.76 (735.71)] with a significant (<0.001) difference from healthy reference. Sclerostin showed a significant positive correlation with fasting blood sugar (r=0.200; p=0.009), HbA1c (r=0.394; p<0.001) and free fatty acids (r=0.205; p=0.007) in total study participants. The SPISE index showed a significant positive correlation (r=0.269, p=0.043) in the prediabetic group. SOST, GLUT4, and insulin receptor (IR) mRNA expression all corroborate with the glycemic status.

Conclusion

Significantly higher expression of sclerostin (both protein and gene) in newly diagnosed T2D and prediabetes male patients, as well as significant association with SPISE index, suggest that sclerostin might be an indicator of pathophysiology related to insulin resistance, which is a characteristic feature of diabetes mellitus. However, the identification of causal relationships would warrant a large-scale prospective cohort study.

## Introduction

Diabetes Mellitus is becoming a global burden as its prevalence rises day by day. Diabetes is now recognized as a major chronic pandemic that affects people of all ethnic backgrounds and socioeconomic statuses, both in developing and developed countries, and in the majority of countries, it has been considered a "public health priority". [1,2]. According to the International Diabetes Federation (IDF), 537 million people worldwide have diabetes, accounting for 10.5 percent of the global population, and this number is expected to climb to 783 million by 2045 [3]. As a well-known fact, in a developing country like India, the majority of diabetes patients face significant out-of-pocket costs. As a result, this sickness not only affects the patient's health, but it also affects the entire family's peace of mind by posing a financial burden due to greater treatment costs Because type 2 diabetes (T2D) is a chronic disorder, individuals usually have a period of modestly raised blood sugar levels before being diagnosed, a condition known as prediabetes [4]. Hence if remains uncared, prediabetes cases might lead to diabetes mellitus.

The etiopathology of T2D is complex and yet to be elucidated in detail. The Wnt signaling pathway plays a crucial role in a variety of cell functions, including pancreatic-cell activity [5]. Glypicans, R-spondin proteins, Kremen-1, Kremen-2, Norrin, Sclerostin (SOST), and MESDC2 are among few of the proteins recognized as modulators of the Wnt signaling pathway. Sclerostin is a 190-residue secreted glycoprotein expressed by the SOST gene on chromosome 17q12-q21 in humans [6]. Sclerostin is largely secreted by osteocytes, however, it is also found in other tissues, and it inhibits bone growth by acting as an anti-anabolic agent [7]. Sclerostin has recently been discovered to bind to LRP5/6 receptors and block the Wnt signaling pathway [8,9]. Bone development is reduced when the Wnt pathway is inhibited [9]. Increased sclerostin levels and a link between sclerostin levels and insulin resistance in skeletal muscle, liver, and adipose tissue were investigated in a cross-sectional study of people with prediabetes only [10]. In another cross-sectional study, sclerostin was found to be increased in T2D patients and a relationship between sclerostin levels with the duration of T2D and HbA1c was documented [11]. Thus, findings from the aforementioned research studies suggest that Wnt signaling has an association with glucose homeostasis. However, no study has been conducted including all three groups (healthy, prediabetes, and diabetes). Hence, the goal of this study was to see if there was any link between sclerostin, glycaemic parameters, and insulin resistance/sensitivity in Indian individuals with diabetes and prediabetes, which was not addressed before.

## Materials and methods

Study design

A total of 171 study participants were included in this clinic-based observational study, conducted at the Department of Biochemistry, All India Institute of Medical Sciences, Rishikesh, Uttarakhand, India. 114 adult males with newly diagnosed T2D and prediabetes (57+57) were invited to participate. Diabetes and prediabetes cases were diagnosed based on the American Diabetes Association (ADA) criteria. 57 Healthy male individuals aged 18-70 years were also recruited. Individuals having one of the following criteria were excluded from the study: if they were female (to avoid the influence of hormonal changes) or had type 1 diabetes, compromised renal function, malignant tumor, bone disease, cardiovascular disease, or other diseases influencing insulin sensitivity and bone health.

Study procedure and data collection

After taking consent from all study participants, anthropometric measurements like height, weight, body mass index, waist circumference, hip circumference, waist-hip, ratio, and BMI were carried out. Then blood samples were collected in ethylenediaminetetraacetic acid (EDTA), sodium fluoride (NaF), and plain vials. Blood investigations included blood sugar levels, glycated haemoglobin (HbA1c), lipid profile, high sensitivity C-reactive protein (hsCRP), calcium, insulin, parathyroid hormone (PTH), free fatty acids (FFA), and sclerostin. The sclerostin level was measured by an enzyme-linked immunosorbent assay (ELISA) kit (Boster Bio, Pleasanton, USA) as per the manufacturer’s protocol.

Homeostasis model assessment-estimated insulin resistance (HOMA-IR= fasting insulin (microU/L)×fasting glucose (nmol/L)/22.5) [12], quantitative insulin-sensitivity check index (QUICKI=1/(log(fasting insulin in μU/ml)+log(fasting glucose in mg/dl)) [13], single point insulin sensitivity estimator (SPISE= 600×HDL^0.185^/(TG^0.2^×BMI^1.338^)) [14], homeostasis model assessment of β-cell function (HOMA-B= 20×fasting insulin (μIU/ml)/ fasting glucose (mmol/ml)-3.5) [15], and adipose tissue insulin resistance index (Adipo-IR = fasting insulin (pmol/L)×free fatty acids (mmol/L)) [16] were calculated using the formula recommended by previous studies [12-16].

RNA was extracted using the TRIzol® method (Invitrogen, Waltham, USA) from all study samples and quantification was done using the NanoQuant Plate™ with Tecan infinite F200pro multi-mode reader (Tecan, Männedorf, Switzerland). RNA with a 260/280 ratio of 1.7-2.0 was considered pure RNA. 500 ng RNA was used to synthesize complementary (cDNA) using a cDNA reverse transcription kit (Applied Biosystems, Waltham, USA) as per the manufacturer’s protocol. Primers were designed using Primer-BLAST (National Center for Biotechnology Information),** **an online primer designing tool​​​​ (https://www.ncbi.nlm.nih.gov/tools/primer-blast/). Primer sequences are provided in Table 1. Real-time polymerase chain reaction (PCR) was carried out on a CFX96 Touch Real-Time PCR System (Bio-Rad, Hercules, USA) with the SYBR Green reagent. The data were normalized with expression CT values of housekeeping gene β-actin and the expression fold change for each sample was calculated using the 2^(-ΔΔCT) method [17].

**Table 1:** Primer sequences for qRT-PCR GLUT4 - glucose transporter type 4, IR - insulin receptor, SOST- sclerostin gene, qRT-PCR - real-time quantitative reverse transcription polymerase chain reaction

Primer Name	Sequence (5’ to 3’)
β-Actin forward primer	GCATGGGTCAGAAGGATTCCTA
β-Actin reverse primer	TGTAGAAGGTGTGGTGCCAGAT
GLUT4 forward primer	GAATCCCTGCAGCCTGGTAG
GLUT4 reverse primer	GTCACACGAGGGGAATGAGG
IR forward primer	CGAGAAGACCATCGACTCGG
IR reverse primer	GACACCAGAGCGTAGGATCG
SOST forward primer	ACACAGCCTTCCGTGTAGTG
SOST reverse primer	GGTTCATGGTCTTGTTGTTCTCC

Statistical analysis

SPSS version 23 (IBM Corp., Armonk, USA) was used to carry out statistical analysis. Data are presented as mean ± standard deviation (SD) or median (interquartile range, IQR) depending on the normality test. Kruskal-Wallis or one-way ANOVA with Bonferroni correction was used to see any differences among groups. Mann-Whitney U test and independent t-test were used for the analysis of the two groups. Pearson’s correlation test was used for correlating sclerostin with different clinical parameters. A p-value <0.05 was considered significant.

## Results

**Figure 1:**
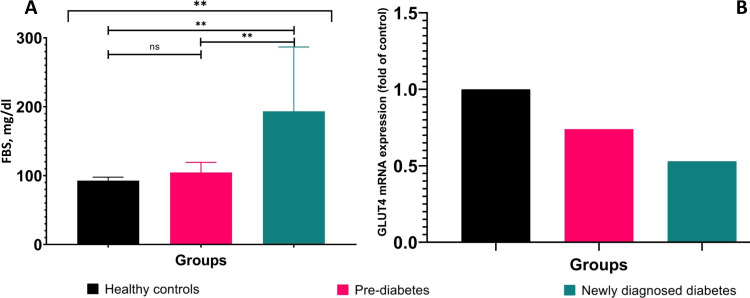
Graphical representation of fasting blood sugar and GLUT4 mRNA expression among healthy controls, prediabetes and newly diagnosed type 2 diabetes ** denotes p<0.001, FBS - fasting blood sugar, GLUT4 - glucose transporter type 4, mRNA - messenger RNA

There was no significant difference among groups with respect to height, weight, and BMI. No age difference was found between prediabetes (46.42±11.43) and newly diagnosed T2D patients (46.02±10.70). Individuals with prediabetes and newly diagnosed T2D were older than healthy individuals. As expected, the newly diagnosed T2D patient group had significantly (p<0.001) elevated fasting blood sugar levels than the healthy control group (Figure 1A). Moreover, when the prediabetes and the newly diagnosed T2D group were compared to the healthy control group, it was observed that their post-prandial blood sugar (PPBS) and HbA1c levels were significantly (p<0.001) elevated. The demographic and biochemical parameters are presented in Table 2.

**Table 2:** Demographic and biochemical parameters of the study participants Data are presented in mean ± SD and median (IQR), IQR - interquartile range, BMI - body mass index, FBS - fasting blood sugar, PPBS - post prandial blood sugar, PTH - parathyroid hormone, HOMA-IR - homeostasis model assessment-estimated insulin resistance, QUICKI - quantitative insulin sensitivity check index, SPISE index - single point insulin sensitivity estimator index, HOMA-B - homeostasis model assessment of β-cell function, Adipo-IR - adipose tissue insulin resistance index, HDL - high-density lipoprotein, LDL - low-density lipoprotein, hsCRP - high sensitivity C-reactive protein, HbA1C - glycated haemoglobin

Parameters	Healthy (n=57)	Pre-diabetes (n=57)	T2DM (n=57)	Total (n=171)	p-value
BMI (kg/m^2^)	24.94 ± 3.55	25.41 ± 3.42	26.55 ± 6.03	25.63 ± 4.52	0.147
FBS (mg/dl)	92.71 ± 5.10	104.53 ± 14.69	193.34 ± 93.37	130.19 ± 70.57	<0.001
PPBS (mg/dl)	105.70 ± 10.67	156.56 ± 13.70	194.19 ± 104.95	185.49 ± 100.52	<0.001
HbA1c (%)	5.40 (0.30)	6.00 (0.40)	9.00 (3.75)	6.0 (1.8)	<0.001
Total Cholesterol (mg/dl)	203.44 ± 37.88	189.03 ± 40.30	186.12 ± 62.95	192.86 ± 48.69	0.126
Triglycerides (mg/dl)	128.0 (78.0)	163.0 (125.0)	162.0 (145.50)	144.0 (109.0)	0.002
HDL (mg/dl)	49.25 ± 10.03	45.54 ± 11.57	39.26 ± 11.19	44.68 ± 11.64	<0.001
LDL (mg/dl)	126.95 ± 26.32	114.11 ± 26.43	116.23 ± 42.08	119.09 ± 32.77	0.080
HsCRP (mg/L)	1.0 (1.71)	1.20 (2.91)	3.1 (4.81)	1.53 (3.3)	<0.001
Calcium (mg/dl)	9.39 ± 0.43	9.32 ± 0.56	9.33 ± 0.65	9.34 ± 0.55	0.781
Insulin (mU/L)	9.70 (6.66)	10.62 (13.05)	9.62 (9.75)	9.78 (9.84)	0.181
PTH (pg/ml)	62.55 ± 23.70	63.06 ± 24.18	60.98 ± 30.56	62.19 ± 26.19	0.908
Free Fatty Acids (mmol/ml)	1.97 (1.05)	2.17 (2.63)	5.57 (4.21)	2.35 (3.66)	<0.001
Sclerostin (pg/ml)	242.12 (158.44)	256.06 (299.65)	465 (735.71)	281.92 (311.23)	<0.001
HOMA-IR	2.32 (1.47)	3.03 (3.69)	3.75 (4.35)	2.70 (3.6)	<0.001
QUICKI	0.34 (0.04)	0.32 (0.06)	0.31 (0.05)	0.33 (0.06)	<0.001
SPISE	6.59 ± 1.61	6.13 ± 1.51	5.49 ± 1.59	6.07 ± 1.63	<0.001
HOMA-B	108.00 (94.97)	94.70 (188.33)	32.02 (44.08)	73.89 (102.60)	<0.001
Adipo-IR	123.02 (125.52)	239.65 (320.02)	358.79 (396.28)	188.60 (320.90)	<0.001

Glucose is taken up in muscles with the help of the GLUT4 receptor. To understand the reason for hyperglycemia, the expression of GLUT4 was investigated. GLUT4 mRNA expression was seen to decrease in prediabetes (0.74-fold) and newly diagnosed T2D (0.53-fold) as compared to healthy controls (Figure 1B).

**Figure 2:**
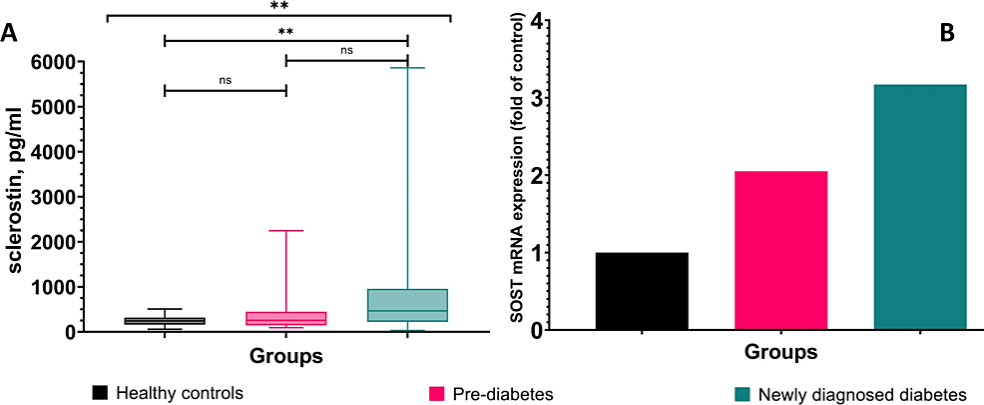
Graphical representation of circulating sclerostin and SOST mRNA expression comparison among healthy controls, prediabetes and newly diagnosed type 2 diabetes ** denotes p<0.001, SOST - sclerostin gene

Sclerostin was significantly (p<0.001) higher in T2D (465.76 (735.71)) and non-significantly elevated in prediabetes group (256.06 (299.65)) compared to healthy (251.05 (158.44)) (Figure 2A). SOST mRNA expression showed 2.05-fold higher in prediabetes and 3.17-fold in newly diagnosed T2D compared to healthy controls (Figure 2B).

**Figure 3:**
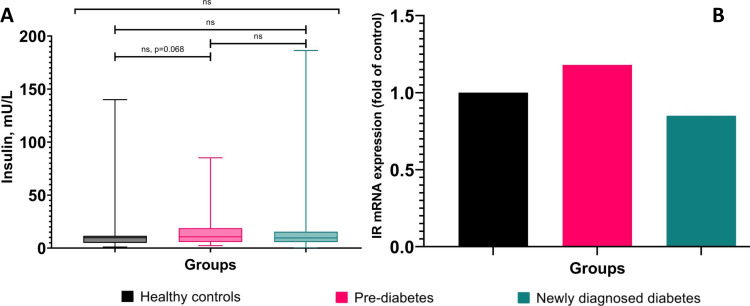
Graphical representation of fasting insulin and insulin receptor (IR) mRNA expression among healthy controls, prediabetes and newly diagnosed type 2 diabetes ns: non-significant, mRNA - messenger RNA

Triglycerides (TG) and free fatty acids (FFA) were found significantly (p<0.05) elevated in prediabetes and the newly diagnosed T2D group compared to the healthy control group. HsCRP was found significantly (p<0.001) elevated only in the newly diagnosed T2D group compared to the healthy control group. However, HDL was found significantly (p<0.001) decreased in the newly diagnosed T2D group compared to the control group. However, total cholesterol, low-density lipoprotein (LDL), calcium, and parathyroid hormone (PTH) did not show any significant difference among the three groups (Table 2). Although there is no significant difference in insulin level among the three groups there is a slightly higher level of insulin in prediabetes cases compared to healthy individuals. Since insulin exhibits its effect through its receptor, we investigated the gene expression of the insulin receptor (IR), which was found to be 1.18-fold higher in prediabetes cases and 0.85-fold in the newly diagnosed T2D group compared to the control group (Figure 3).

Insulin resistance, insulin sensitivity, beta-cell function, and adipose tissue insulin resistance

Inadequate response of muscle cells, hepatic cells, and adipocytes to insulin gives rise to insulin resistance. There are various surrogate markers for measurement of insulin resistance/sensitivity such as HOMA-IR, QUICKI index, SPISE index, and Adipo-IR. The HOMA-IR values were found significantly elevated (p<0.001) in the newly diagnosed T2D group and in the prediabetes group compared to the healthy control group. On the other hand, QUICKI values were found significantly (p<0.001) decreased in the newly diagnosed T2D group and in the prediabetes group compared to the healthy control group. However, there is a gradual decrease in the SPISE index (which is an indicator of whole-body insulin sensitivity and calculated based on TG, high-density lipoprotein (HDL), and BMI values) from healthy to prediabetes and newly diagnosed T2D cases. SPISE index and HOMA-B values were found significantly (p=0.001 and p<0.001) decreased only in the newly diagnosed T2D group compared to the healthy control group. Similarly, there was a gradual increase in Adipo-IR (which is an indicator of response to the glycemic status from adipose tissue) values among healthy, prediabetes, and newly diagnosed T2D cases. Moreover, both prediabetes and newly diagnosed T2D cases showed significant differences (p=0.001 and p<0.001) compared to the healthy control group (Table 2).

Association of circulating sclerostin with glycemic parameters, free fatty acids, PTH, insulin, insulin resistance, insulin sensitivity, β-cell function, and adipose tissues insulin resistance

**Figure 4:**
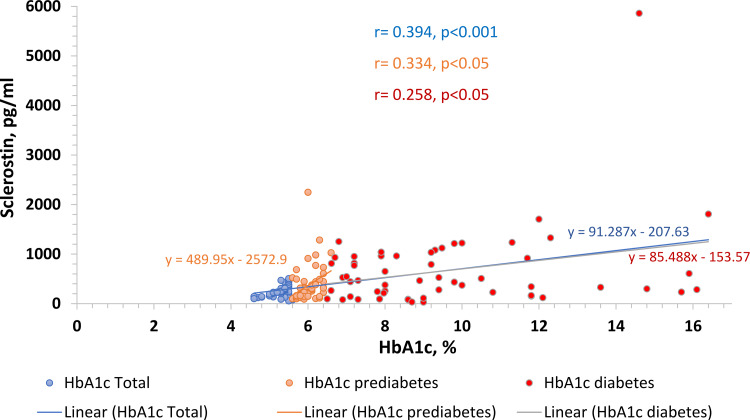
Association between sclerostin with HbA1c among groups HbA1C - glycated haemoglobin

To identify the factors related to circulating sclerostin, Pearson’s correlation test was performed. When all the study participants were considered, circulating sclerostin showed a significant positive correlation with fasting blood sugar (r=0.200, p=0.009), HbA1c (r=0.394, p<0.001), PPBS (r=0.267, p<0.001) and FFA (r=0.205, p=0.007), However, almost all above-mentioned parameters lost significance when the three groups were taken into account separately, with the exception of the HbA1c level in the healthy control (r=0.438, p=0.001), prediabetes group (r=0.334, p=0.011), and in the newly diagnosed T2D group (r=0.258, p=0.05) (Figure 4).

Moreover, it showed a positive trend with HOMA-IR (r=0.009, p=0.909), Adipo-IR (r=0.023, p=0.766), QUICKI (r=0.043, p=0.573), and a showed negative trend with insulin (r=-0.034, p=0.663) and with HOMA-B (r=-0.101, p=0.191). Furthermore, PTH (r=0.101, p=0.190) showed positive trend with sclerostin, although it was non-significant.

**Figure 5:**
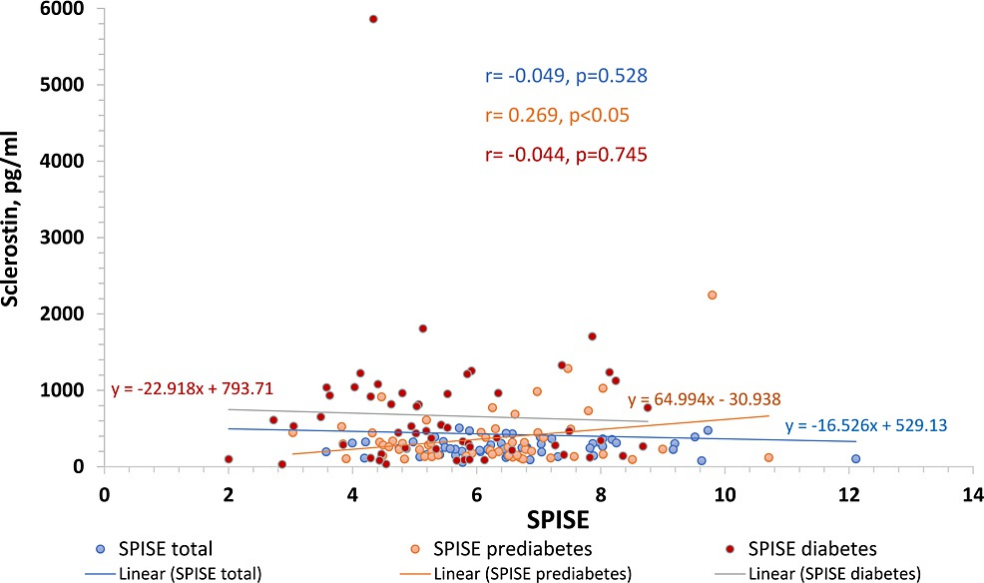
Association between sclerostin and SPISE SPISE index - single point insulin sensitivity estimator index

When all study participants were considered, the SPISE index (r=-0.049, p=0.528) showed no significant association while three groups were taken into account separately, sclerostin revealed a significant (r=0.269, p=0.043) correlation with SPISE index in the prediabetes group but not in the newly diagnosed T2D group (r=-0.044, p=0.745) (Figure 5).

## Discussion

In this study, it was aimed to check the association of sclerostin with insulin resistance/sensitivity, and β-cell function in healthy, prediabetes, and type 2 diabetic patients. There are very few studies worldwide that showed the correlation of sclerostin with insulin resistance in diabetic patients [18,19]. We found significantly higher circulating sclerostin levels in the newly diagnosed type 2 diabetes male individuals compared to the healthy male control group and it was corroborating with SOST mRNA expression (Figure 2B). This finding is consistent with previous findings. [9,10]. Moreover, our study showed a significant positive correlation of sclerostin with fasting blood sugar, PPBS, HbA1c, and free fatty acids, which is consistent with another previous study which found a significant association of sclerostin with fasting blood sugar in a cohort study of 1778 study participants without type 2 diabetes and followed for the relationship between sclerostin and incident of type 2 diabetes, but they did not investigate the correlation between HbA1c and free fatty acids with sclerostin [19] and suggests that sclerostin has a significant association with diabetes mellitus. The gradual decrease in GLUT4 expression supports the altered glycemic parameters and explains the pathology behind hyperglycemia in prediabetes and T2D individuals. Many previous studies showed an already negative relationship between GLUT4 mRNA expression with fasting blood sugar and with HbA1c [20]. Li Q et al (2021) showed in their cohort study that free fatty acids are significantly correlated with higher fasting blood sugar, HbA1c, and PPBS and thus a higher risk of incident diabetes [21]. Free fatty acids are also responsible for inhibiting GLUT4 protein translocation into muscle [22]. A significant association between sclerostin, FFA, and other glycaemic parameters as well as a decrease in GLUT4 mRNA expression provides an insight into the impact of sclerostin on the etiology of altered glucose homeostasis in diabetes mellitus, although the present study design does not provide causal evidence.

A recent study showed that triglycerides alone are capable to affect both insulin resistance and β-cell function [23]. They found triglycerides positively correlated with insulin resistance and negatively correlated with β-cell function. An Indian study has shown that the SPISE index, which is calculated based on TG and HDL values, is a better indicator for IR in the Indian population [24]. Moreover, Verma et al have evidenced that sclerostin had a significant association with apolipoprotein A-I (ApoA1) and apolipoprotein B100 (ApoB100)** **in the Indian population with coronary artery disease [25]. Hence, a significant association of sclerostin in the prediabetes group with the SPISE index, the indicator for whole-body insulin sensitivity calculated based on lipid profile instead of insulin level, indicates that sclerostin, a Wnt signaling inhibitor, may be linked to insulin sensitivity in prediabetic Indian population.

A recent study on obese and lean women showed that higher sclerostin is not associated with insulin sensitivity in lean women [26]. But, the study had only female participants so we could not compare our study findings with the aforementioned study as our study includes only male patients.

In our study, we could not find any significant difference in PTH among the three groups (Table 2) as well as no significant association between sclerostin and PTH levels. García-Martín et al. (2012), showed in a cross-sectional study that PTH level is significantly lower in T2D than in the healthy control group and inversely correlated with sclerostin in T2D group and healthy control group [11]. However, Gennari et al. (2012), showed in their study that sclerostin was negatively associated with PTH in the control group but, a positive nonsignificant trend between PTH and sclerostin in the T2D group [27]. However, both studies had included longer duration (>13.5 years) T2D patients as well as both male and female patients, whereas, in our study, we included only newly diagnosed type 2 diabetes male patients, which may be the reason for the discrepancy in findings observed in our study with previous study results.

Study limitations

Our study has some strengths as well as limitations. To the best of our knowledge, this study represents the first findings on the association of circulating sclerostin with insulin resistance/ sensitivity and β- cell function in the diabetic and prediabetic male Indian population. Since this study involved only male patients, its results cannot be generalized. Since it is a cross-sectional study it does not provide information related to the causative relationship between sclerostin and insulin resistance/sensitivity. The limited sample size in each group and the study design of this study demand a study with a larger sample size and a prospective cohort study.

## Conclusions

In conclusion, this study showed that PPBS, HbA1c, free fatty acids, HOMA-IR, and Adipo-IR significantly increased and QUICKI significantly decreased in prediabetes and newly diagnosed type 2 diabetes but fasting blood sugar and a gradual decline in SPISE index among the three groups. HbA1c shows a significant correlation with sclerostin in prediabetes and newly diagnosed type 2 diabetes. Moreover, the SPISE index showed significant association with sclerostin only in the prediabetes group. The mRNA expression for SOST, GLUT4, and insulin receptor (IR) was also corroborated with glycemic status among all study groups. Hence from this study, it could be concluded that sclerostin might play a crucial role in the modulation of glycemic homeostasis along with insulin resistance in prediabetic individuals. However, it demands a large-scale prospective study to establish the causal relationship.
